# Supplementary material for: Causal Relationship between Aging and Anorexia Nervosa: A White-Matter-Microstructure-Mediated Mendelian Randomization Analysis
Source: Biomedicines. 2024 Aug 16;12(8):1874. doi: 10.3390/biomedicines12081874 (PMC11351342; doi:10.3390/biomedicines12081874)
Supplement: Supplementary file 1 [file biomedicines-12-01874-s001.zip › Supplementary Figure/Figure S4/Singe SNP plot of MR analysis for TL to ubm-b-2090.pdf]

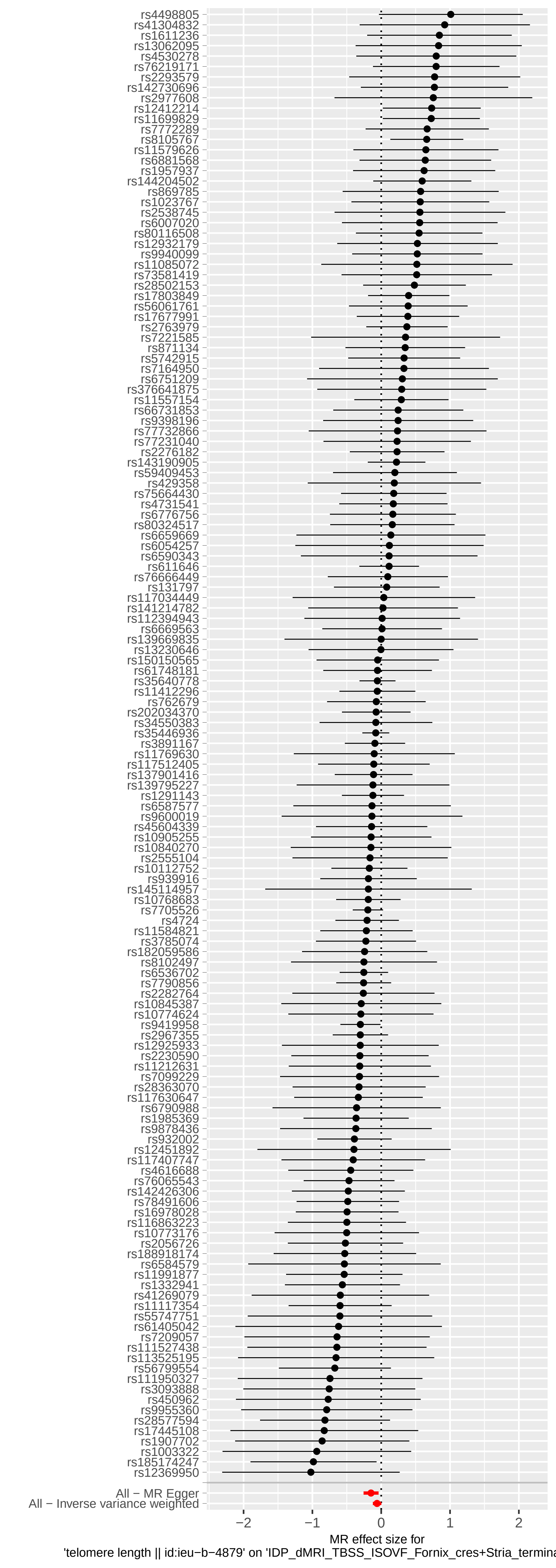

rs4498805
rs41304832
rs1611236
rs13062095
rs4530278
rs76219171
rs2293579
rs142730696
rs2977608
rs12412214
rs11699829
rs7772289
rs8105767
rs11579626
rs6881568
rs1957937
rs144204502
rs869785
rs1023767
rs2538745
rs6007020
rs80116508
rs12932179
rs9940099
rs11085072
rs73581419
rs28502153
rs17803849
rs56061761
rs17677991
rs2763979
rs7221585
rs871134
rs5742915
rs7164950
rs6751209
rs376641875
rs11557154
rs66731853
rs9398196
rs77732866
rs77231040
rs2276182
rs143190905
rs59409453
rs429358
rs75664430
rs4731541
rs6776756
rs80324517
rs6659669
rs6054257
rs6590343
rs611646
rs76666449
rs131797
rs117034449
rs141214782
rs112394943
rs6669563
rs139669835
rs13230646
rs150150565
rs61748181
rs35640778
rs11412296
rs762679
rs202034370
rs34550383
rs35446936
rs3891167
rs11769630
rs117512405
rs137901416
rs139795227
rs1291143
rs6587577
rs9600019
rs45604339
rs10905255
rs10840270
rs2555104
rs10112752
rs939916
rs145114957
rs10768683
rs7705526
rs4724
rs11584821
rs3785074
rs182059586
rs8102497
rs6536702
rs7790856
rs2282764
rs10845387
rs10774624
rs9419958
rs2967355
rs12925933
rs2230590
rs11212631
rs7099229
rs28363070
rs117630647
rs6790988
rs1985369
rs9878436
rs932002
rs12451892
rs117407747
rs4616688
rs76065543
rs142426306
rs78491606
rs16978028
rs116863223
rs10773176
rs2056726
rs188918174
rs6584579
rs11991877
rs1332941
rs41269079
rs11117354
rs55747751
rs61405042
rs7209057
rs111527438
rs113525195
rs56799554
rs111950327
rs3093888
rs450962
rs9955360
rs28577594
rs17445108
rs1907702
rs1003322
rs185174247
rs12369950
All − MR Egger
All − Inverse variance weighted
−2
−1
0
1
2
MR effect size for
'telomere length || id:ieu−b−4879' on 'IDP_dMRI_TBSS_ISOVF_Fornix_cres+Stria_termina
